# 50‐kHz ultrasonic vocalizations do not signal social anhedonia in transgenic DISC1 rats

**DOI:** 10.1002/brb3.2984

**Published:** 2023-04-05

**Authors:** Mohammad Seidisarouei, Sandra Schäble, Marijn van Wingerden, Svenja V. Trossbach, Carsten Korth, Tobias Kalenscher

**Affiliations:** ^1^ Social Rodent Lab and Comparative Psychology, Institute of Experimental Psychology Heinrich‐Heine University Düsseldorf Germany; ^2^ Comparative Psychology, Institute of Experimental Psychology Heinrich‐Heine University Düsseldorf Germany; ^3^ Department of Cognitive Science and Artificial Intelligence, Tilburg School of Humanities and Digital Sciences Tilburg University Tilburg The Netherlands; ^4^ Department of Neuropathology, Medical Faculty Heinrich‐Heine University Düsseldorf Germany

**Keywords:** DISC1, 50-kHz USVs, social reward, non‐social reward, social anhedonia

## Abstract

Patients diagnosed with neuropsychiatric disorders, such as autism and schizophrenia, suffer from disorganized speech. The disrupted‐in‐schizophrenia 1 (DISC1) protein pathway is considered a risk factor for the development of several psychiatric disorders and plays an important role in the dysregulation of dopamine (DA), which in turn plays an important role in the regulation of ultrasonic vocalizations (USVs) in rats. Moreover, the DISC1 protein pathway has been identified as a cause of social anhedonia, that is, a decrease in the drive for social interactions. USVs transmit specific affective information to other rats, with 50‐kHz calls indicating a positive affective state in rats. Dysregulation of the dopaminergic system impacts the qualitative and quantitative features of USVs, such as duration, peak frequency, and the call rate. In this study, we thus used a well‐established transgenic DISC1 (tgDISC1) rat line to investigate whether the neural (decreased DA levels in the dorsal striatum, amygdala, and hippocampus (HPC)) and behavioral (social anhedonia) features of tgDISC1 rats could be manifested through the modulation of their 50‐kHz USVs. Analyses of three features (call rate, duration, and peak frequency) of all 50‐kHz revealed no significant differences between groups, suggesting decreased DA levels in the dorsal striatum and amygdala, and HPC may affect social interaction but leave 50‐kHz USV production intact.

## Graphical abstract

In this article, we sought to determine whether the dysregulation of dopamine and social anhedonia of a tgDISC1 rat model could be detected by their 50‐kHz USVs

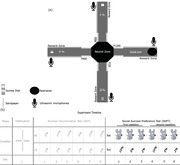

## 1 INTRODUCTION

The disrupted‐in‐schizophrenia 1 (DISC1) gene was initially identified in a Scottish family with an unusually high prevalence of mental disorders, including schizophrenia, and the disruption was due to a balanced translocation of the chromosome (1:11) (q43, q21) (Millar et al., 2001). The DISC1 protein signaling pathway has been linked to multiple deficits in brain development both in humans and animals, which may lead to schizophrenia, bipolar disorder, recurrent major depression, and other neuropsychiatric disorders in humans, as well as phenotypical alterations reminiscent of human psychiatric disorders in animals (Austin et al., 2003; Clapcote et al., 2007; Hashimoto et al., 2006; Kirsty Millar et al., 2000; Shokouhifar et al., 2019). Recently, several studies have shown that the neural dysregulation caused by DISC1 impairs the Dopamine (DA) system by increasing the affinity of DA‐D2 receptors and increasing the removal of DA from the synaptic cleft because of translocation of the DA transporter, resulting in decreasing DA levels in the dorsal striatum, amygdala, and hippocampus (HPC, Hennah & Porteous, 2009; Ripke et al., 2014; Trossbach et al., 2016; Wang et al., 2017). In a recent study (Seidisarouei et al., 2022), we compared the choice behavior of transgenic DISC1 (tgDISC1) rats (Klein & Platt, 2013; Seidisarouei et al., 2022; Wang et al., 2022) with that of wild‐type (WT) control rats in a novel reward paradigm in which animals could choose between two types of reinforcers, an opportunity for social interaction (social reward) versus consumption of sucrose solution (nonsocial reward). tgDISC1 rats showed a significantly reduced interest in social interaction but a similar preference for sucrose consumption, compared to WT rats. In other words, tgDISC1 rats spent significantly less time interacting with a juvenile conspecific, which may resemble social anhedonia, that is, the decreased interest in potentially rewarding social activities (Chapman et al., 1976), seen in patients with depression or schizophrenia (American Psychiatric Association Diagnostic and Statistical Manual of Mental Disorders [DSM‐IV], 2012). This social anhedonia in tgDISC1 rats, most likely caused by DA dysregulation, may also manifest in rat vocal communication, as DA also plays an important role in the processing and production of rat 50‐kHz USVs (Burgdorf & Knutson, 2001). In support of this idea, it has been reported that rats with reduced social motivation vocalized fewer 50‐kHz USVs (Riaz et al., 2015) and rats that selectively bred to low levels of 50‐kHz USVs showed significant changes in their social interactions (Harmon et al., 2008). Presumably, a deficit in USV expression and perception might interrupt the natural back‐and‐forth of social communication, thereby reducing social interest or motivation.

In recent years, a body of convergent studies demonstrated the implication and importance of USVs by rats in representing their emotional and motivational states.

In terms of the frequency at which USVs are emitted, they can broadly be categorized as 22 and 50 kHz. The USVs of these two distinct families (22 and 50 kHz) signal aversive and appetitive qualitative information, respectively, about the rats’ affective states that may be triggered by either social or nonsocial stimuli that possess affective valence. Rats emit 22‐kHz USVs under aversive conditions such as fear, pain, and danger (Sadananda et al., 2008; Wöhr & Schwarting, 2010), whereas they emit 50‐kHz calls in response to or anticipation of appetitive stimuli, such as playing, social interaction, eating, mating, and administration of drugs with rewarding properties (Bialy et al., 2000; Brudzynski & Pniak, 2002; Mulvihill & Brudzynski, 2018; Simola & Granon, 2019). Accordingly, playback of prerecorded appetitive 50‐kHz USVs induces social approach behavior that paves the way for social interactions, supporting the idea that 50‐kHz USVs serve as social contact calls to (re)establish or maintain contact between rats (Kalenscher et al., 2020; Wöhr & Schwarting, 2012). Because of these properties, USVs are believed to have substantial adaptive value for the survival and well‐being of rats as a social species (Wöhr & Schwarting, 2013).

To better understand and more clearly interpret rat USV, it is fundamental to uncover its neural basis. In this regard, studies demonstrated a fundamental role of dopaminergic neurotransmission in USV production. For example, the DA agonist apomorphine (by acute systemic injection) can promote 50‐kHz calls (Williams & Undieh, 2010), and the D2/D3 agonist quinpirole (by intra‐NcAcc administration) modulates USV production (Brudzynski et al., 2012). Conversely, DA receptor antagonists prevented the expected emission of 50‐kHz USVs by various rewards (natural and artificial), such as systemic cocaine (Williams & Undieh, 2010), intracerebral amphetamine (AMPH) (Thompson et al., 2006), tickling, electrical brain stimulation (Burgdorf et al., 2007), and mating contexts (Bialy et al., 2010; Ciucci et al., 2007). Furthermore, DA agonists or antagonists cause not only changes in quantity but also the quality of 50‐kHz USVs. For example, haloperidol is a D2 receptor antagonist, reduces the bandwidth, amplitude, and complexity of 50‐kHz calls, similar to the effects of a unilateral infusion of 6‐hydroxydopamine (6‐OHDA, Ciucci et al., 2009, 2007). In addition to the decreased call rate and altered call profile, antagonism of D1 and D2 receptors alone or combined altered several features of 50‐kHz calls, such as duration, amplitude, and latency to call (Ringel et al., 2013; Wright et al., 2013).

Thus, 50‐kHz USVs are associated with appetitive social and nonsocial situations and DA. We can exploit this property to quantify the expected and experienced value that rats attribute to a reward, including social contact (Heyse et al., 2015; Knutson et al., 1998, 1999). Therefore, here, we investigated whether changes in patterns of 50‐kHz USVs emission accompany the social anhedonia expressed in tgDISC1 rats. To this end, we analyzed different quantitative and qualitative characteristics of the 50‐kHz USVs, such as call rate, duration, and peak frequency, in rats performing a social decision task in which they chose between social and nonsocial rewards.

## 2 MATERIALS AND METHODS

### 2.1 Subjects

The animal experiment was permitted by the local authorities (Landesamt für Natur, Umwelt und Verbraucherschutz North Rhine‐Westphalia, Germany) and conducted according to the European Union Directive 2010/63/E.U. The findings that, compared to WT rats, tgDISC1 rats are less motivated to socially interact with juvenile conspecifics, but have comparable preferences for sucrose rewards, have been published before (Seidisarouei et al., 2022), based on the current sample of animals. tgDISC1 Sprague Dawley rats and their sibling WT littermate controls were bred at the local animal facility (ZETT, Heinrich‐Heine University, Düsseldorf, Germany). In total, we used 36 male Sprague Dawley rats for our study. The rats were divided into three groups: (1) tgDISC1 group (*n* = 12 rats), weighing *m* = 285 g and aged 57–60 days at the beginning of the Social‐Sucrose Preference Test (SSPT), serving as the actor rats; (2) WT group (*n* = 12 rats), weighing *m* = 304 g and aged 57–60 days at the beginning of the SSPT, serving as the actor rats; and (3) a juvenile WT group (*n* = 12 rats), weighing *m* = 145 g and aged 28–30 days, serving as the social stimuli. The tgDISC1 rats were bred through the identical method introduced by Trossbach et al. (2016). Experimental rats were kept in groups of *n* = 2 for actors and *n* = 3 for social stimulus rats in standard Type IV Makrolon cages in a reversed 12:12 h light–dark cycle. The stable room was kept constantly at a temperature of 22°C±2 and a humidity of 55% ±2. All actor rats received standard laboratory rodent food, ad libitum.

### 2.2 Behavioral task

**FIGURE 1:**
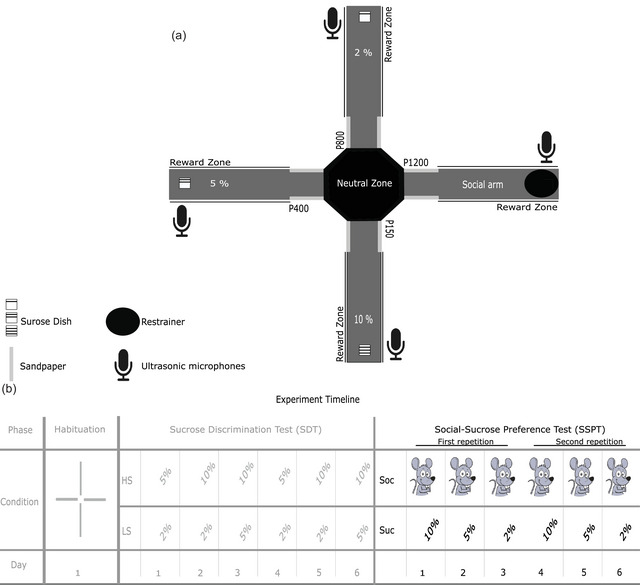
Design of the X‐shaped chambered sociability test (XCST). (**A)** Schematic diagram of the XCST maze showing the positions for the nonsocial reward, microphones, the restrainer for the social reward, and the positions and gradations of the sandpaper (P150, P400, P800, and P1200). Part (**B)** shows an example of the schedule of the experiment for different phases, days, and conditions. Habituation: examination of the free arm in the habituation phase, sucrose discrimination test (SDT): HS; higher sucrose in a given trial, LS; lower sucrose in a given trial, social‐sucrose preference test (SSPT): Soc; social reward, and Suc; sucrose. We show all details of the experiment for the sake of completion, but the grayed‐out parts of the table refer to task phases reported elsewhere (Seidisarouei et al., 2022); here, we only report data obtained from the phases in the black part of the table.

The USV data analyzed in this study were recorded from the SSPT (see later). SSPT was the final phase of a behavioral study published recently (Seidisarouei et al., 2022), and the so‐called X‐shaped chambered sociability test (XCST, see Figure 1A, Seidisarouei et al., 2021). The XCST task is designed to detect differences in preference for two types of rewards, social reward (interaction with the social stimulus rat) and nonsocial reward (consumption of liquid rewards with either 2%, 5%, or 10% sucrose concentration). The XCST consists of three phases: Habituation, Sucrose Discrimination Test (SDT), and SSPT. The habituation phase aimed to determine whether animals have an inherited bias for, or against, any of the features used in setup or apparatus, such as a side‐bias (Figure 1A). In the second phase, SDT, the goal was to determine whether animals can discriminate between the three sucrose concentrations (2%, 5%, and 10%) used as nonsocial rewards. In the SSPT, animals chose simultaneously between nonsocial reward and social reward. To this end, rats were trained in a 4‐arm plus maze in which sandpapers of different gradations marked the entrance of the arms. Each arm was baited with one of the three sucrose rewards, or the social stimulus rat, with the arm‐reward contingency randomized across rats (for details, see Seidisarouei et al., 2021). The SSPT comprised three choice conditions, social reward versus 2% sucrose, social reward versus 5% sucrose, and social reward versus 10% sucrose (Figure 1A). On each testing day, two of the four arms in the XCST maze were open, and the other two were closed. At the beginning of each test, rats were placed in the center of the maze, and they could choose to explore both open arms for 8 min, yielding either the social reward or one of the three sucrose rewards, depending on the task condition. All rats underwent two repetitions of all three choice conditions. The order of conditions was pseudo‐randomized across repetitions and rats (Figure 1B).

As previously shown, the value of social interactions declined over time with increasing familiarity between actor and social stimulus rats (Smith et al., 2015, 2017). To prevent this effect from affecting USV production, 12 different social stimulus rats were used to maintain the novelty and value of social interaction across testing sessions in the SSPT. In addition, the social stimulus rats’ assignment was counterbalanced across actor rats.

## 3 BEHAVIORAL ANALYSIS

### 3.1 Video‐tracking

We used EthoVision (XT version 11.5, Noldus) to track the animals’ positions. The arena setting of the SSPT phase was designed to track the animals in reward zones (Figure 1A).

## 4 USVS RECORDING, ANALYSIS, AND LABELING PROCEDURE

### 4.1 Recording

In order to record USVs, four ultrasonic microphones (condenser microphone CM16/CMPA, Avisoft Bioacoustics, Glienecke, Germany) were positioned by a microphone stand at a distance of approximately 20 cm on the right side above each reward dish, and also to perform acoustic analysis of USVs, we used the Avisoft‐SASLab Pro (Version 5.2, Avisoft Bioacoustics, Berlin, Germany). In Avisoft‐SASLab Pro, the spectrograms with a frequency resolution of 390 Hz and a time resolution of 0.64 ms were created by a fast Fourier transformation with a length of 512 points and an overlap of 75% (flat top window, 100% frame size).

### 4.2 Labeling

A trained scorer identified the calls and assigned them either to a 22‐kHz (frequency <30 kHz) or a 50‐kHz (frequency >30 kHz) category. In total, the calls of 144 trials (24 actors × 3 conditions × 2 repetitions) in the SSPT had to be recorded, but due to technical issues, we lost USVs of 33 trials in different conditions of SSPT (juvenile vs. 2%; WT = 2, tgDISC1 = 3, juvenile vs. 5%; WT = 5, tgDISC1 = 8, Juvenile vs. 10%; WT = 8, tgDISC1 = 7). In addition to 50‐kHz calls, rats also vocalized 22‐kHz USVs; however, because the main focus of this study was 50‐kHz calls, we did not include 22‐kHz calls in our analysis.

### 4.3 USV localization

We generated a USV position map that shows where in the maze the individual USV events were emitted, as explained as follows. To reach a time series of vocalization labels with a temporal resolution of 25 Hz, we exported the USV raw data (Avisoft SAS‐Lab Pro's output) and synchronized them to the video stream of rat positions within the maze (Ethovision output). Notably, in behavioral tracking and analyses, only the time spent in reward zones was measured; therefore, we only identified, labeled, and analyzed the 50‐kHz USVs emitted in both reward zones (social and nonsocial).

### 4.4 Software

All statistical analyses ran using SPSS Statistics (version 24; IBM, USA), and figures were created by Jupyter Notebook (Kluyver et al., 2016) through the packages matplotlib (Hunter, 2007), pandas (McKinney, 2010), ptitprince (Allen et al., 2019), and seaborn (Waskom, 2021). To edit the figures, we used Inkscape (Inkscape, 2020).

### 4.5 Acoustic feature analysis

In order to detect between‐group differences, we examined three features of all USVs: call rate (number of calls vocalized per animal in each reward zone/time (s) animal spent in each specific reward zone), duration (s), and frequency (kHz) by conducting three separate two‐way ANOVAs with group (tgDISC1 and WT) as between‐subject factor and reward zone (social and nonsocial; we pooled USV data across all three sucrose zones) as a within‐subject factor. In addition to investigate possible group differences in the relationship between the frequency of calls and the time spent in the social reward zone, or the sucrose reward zone, respectively, we ran mixed linear model analyses for each zone. The frequency of calls was quantified as a number of calls in the respective zone/[calls in the social zone + calls in the sucrose reward zone] × 100.

Finally, as an exploratory analysis (see Supporting Information section, Figure S1, and Table S1), we ran a three‐way ANOVA to find out whether the number of 50‐kHz USVs of groups differed over 8 min (each trial duration). In this analysis, we took group as a between‐group factor, reward zone (social and nonsocial), and time (in full minutes) as within‐subject factors and the mean number of 50‐kHz USV emitted per minute in all conditions and repetitions as the dependent variable. The significance level at *p* < .05 was set for all statistical analyses, and all the post hoc tests were Bonferroni‐corrected for multiple comparisons.

## 5 RESULTS

### 5.1 tgDISC1 rats show reduced interest in social contact

Details of the rats’ choice behavior are described elsewhere (Seidisarouei et al., 2022). Briefly, we found that tgDISC1 rats differed from WT rats in their social, but not in their nonsocial reward preferences: Compared to WT rats, DISC1 rats spent less time in the social reward zone that offered the opportunity to interact with the juvenile conspecific, and more time in the nonsocial reward zones that offered the opportunity to consume sucrose solution. The reduced time spent interacting with the conspecific was unlikely due to a hypersensitivity for sugar solution in the sucrose zones because we found no difference in sucrose preference and sucrose reward‐seeking behavior between tgDISC1 and WT rats in the SDT. We conclude that the reduction in time spent interacting with conspecifics reflects genuinely reduced interest in social contact, that is, social anhedonia.

### 5.2 50‐kHz USVs

#### 5.2.1 Characterization of all 50‐kHz USVs

In total, *n* = 30,092 50‐kHz USVs were identified.

**FIGURE 2:**
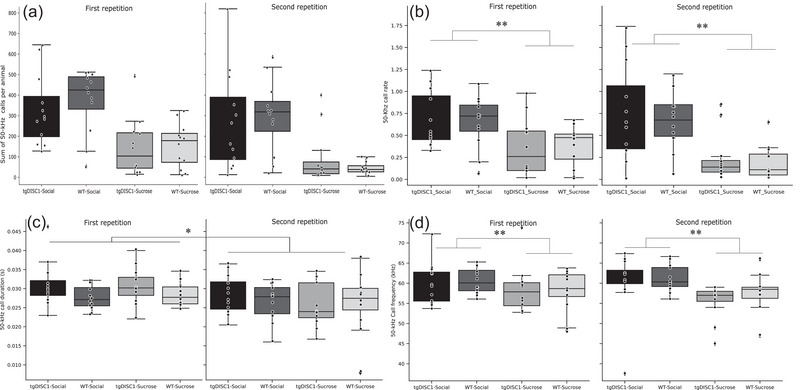
Illustration of the number of ultrasonic vocalizations (USVs) (A), call rate (B), duration (C), and peak frequency (D) of disrupted‐in‐schizophrenia 1 (DISC1) and wild‐type (WT) rats in all reward zones and across both repetitions. *p < .01 **p < .001.

There was large individual variability in vocalization activity between animals in both groups and all reward zones (Figure 2A).

#### 5.2.2 Call rate

The three‐way ANOVA did not reveal a between‐group difference in the rate of calls vocalized per group in both reward zones over two repetitions (*F* [1,22] = 0.86, *p* = .77; for more details, see Figure 2B, Table 2A). Likewise, we found no significant results for the within‐subject factor *repetition* (*F* [1,22] = 1.7, *p* = .194, Figure 2B, Table 2B). However, the analysis showed a significant main effect of the reward zone (*F* [1,22] = 65.3, *p* ≤ .001), showing an expected higher rate of calls in the social reward zone than in the sucrose zones (Figure 2B. a and Table 2C). Furthermore, there was an interaction effect of reward zone × repetition (*F* [1,22] = 8.3, *p* ≤ .009, Table 2D), demonstrating that animals produced fewer calls in the second repetition than the first repetition in the sucrose zone. No other interaction effect reached significance.

### 5.3 Call duration

The results of the three‐way ANOVA analysis revealed no significant difference between tgDISC1 and WT rats in the duration of calls (*F* [1,22] = 1.7, *p* = .196, Figure 2C and Table 2E). Moreover, we found no significant difference in the duration of calls between the reward zones (*F* [1,22] = 0.1, *p* = .920, Figure 2C, Table 2F). However, the analysis showed that rats’ calls did have a longer duration in the first repetition than the second repetition (*F* [1,22] = 7.6, *p* = .011, Figure 2C. a, Table 2G). Again, no significant interaction effect was found.

### 5.4 Call peak frequency

Analyzing the peak frequency of calls emitted by rats through a three‐way ANOVA did not yield a significant difference (*F* [1,22] = 0.28, *p* = .597, Figure 2D and Table 2H). There was a significant effect of the within‐subject factor *reward zone* on call peak frequency (*F* [1,22] = 10.4, *p* ≤ .004, Figure 2D. a, Table 2I), showing that animals in the social reward zone vocalized with a higher frequency compared to the sucrose reward zones. The other within‐subject factor, repetition, did not yield a significant effect (*F* [1,22] = 0.301, *p* = .589, Figure 2D, Table 2J). No significant interaction effect was found in this analysis.

### 5.5 Mixed linear model analysis

This analysis showed no significant difference between groups (*β*_i_ = −.62, SE = 0.46, *z* = −1.34, CI [−1.52, 0.28], *p* = .177; for more information see Table 3) in the percentage of 50‐kHz calls vocalized in the social reward zones as a function of percent time spent in the social reward zone.

## 6 DISCUSSION

Our findings did not demonstrate significant between‐group differences in 50‐kHz USV vocalization patterns between tgDISC1‐rats and WT controls. This null effect is inconsistent with our prediction that differences in USVs between tgDISC1 and WT rats would reflect or even mediate, and the difference in social motivation reported earlier (Seidisarouei et al., 2022; Wang et al., 2022). In the following, we will offer a tentative explanation for these null‐results.

### 6.1 Regional specificity of tgDISC1‐induced DA transmission effect

Studies have shown that the dorsal striatum (caudate and putamen) plays a critical role in assigning value to a social object and encoding it as a reward (Acevedo et al., 2012; Clements et al., 2022; Klein & Platt, 2013). In fact, a deficit in dorsal striatum function is associated with low‐value attribution for social interaction in autism (Clements et al., 2022), demonstrating the crucial role of the dorsal striatum in valuing and encoding a social object. On the other hand, findings show a significant role of the ventral striatum (Burgdorf & Knutson, 2001; Mulvihill & Brudzynski, 2019) and not the dorsal striatum (Burgdorf & Knutson, 2001; Costa et al., 2019) in the emission of 50‐kHz USVs. More specifically, although microinjections of DA agonists into the nucleus accumbens shell increased the emission of 50‐kHz USVs (Mulvihill & Brudzynski, 2019), microinjection of AMPH into the dorsal striatum or DA denervation in the dorsal striatum did not result in changes in the number of 50‐kHz USVs (Costa et al., 2019). In this context, findings suggest that 50‐kHz USV can release phasic DA (Willuhn et al., 2014) and that DA release is not always followed by USV production (Simola et al., 2012). On the other hand, DA release in the nucleus accumbens accompanies the perception of 50‐kHz USVs which induce social approach in rats (Willuhn et al., 2014). These findings may suggest that USV production is less DA‐dependent than previously thought. For example, a study by Wright et al. (2013) demonstrated that the frequency of 50‐kHz USVs and the distribution of call subtypes in response to AMPH treatment are linked to the action of DA on D1‐ and D2‐like receptors. However, blocking the reuptake of DA is not enough to trigger the emission of calls. Moreover, at this point, it should be noted that several studies have shown the importance of non‐dopaminergic transmissions such as serotonin (Wöhr et al., 2015), glutamate (Costa et al., 2015; Panksepp & Burgdorf, 2000), norepinephrine (Branchi et al., 2001; Grant et al., 2018), adenosine (Simola et al., 2016), and glucocorticoids (Popik et al., 2014) in the emission of 50‐kHz USVs, indicating that USVs are a compound behavior that does not depend on DA alone. This may explain why tgDISC1 rats do not differ from WT rats in 50‐kHz USV behavior despite their DA deficiency.

Moreover, the reduced DA levels in the amygdala and HPC of tgDISC1 rats may disrupt social interactions (Allsop et al., 2014; Davis et al., 2009; Hernandez‐Lallement et al., 2016), but not the production of 50‐kHz USVs. As shown, the amygdala is involved in the perception of 50‐kHz USVs and social approach behavior (Schönfeld et al., 2020), but to the best of our knowledge, there is limited research on the role of the amygdala in the production of 50‐kHz USVs. Similarly, the HPC's role in 50‐kHz USV production remains unknown.

In addition, previous research has demonstrated that prior experience can reduce the duration of rat USVs (Wöhr et al., 2008) and this might be the reason why we detected a longer call duration in the first compared to the second task repetition in the SSPT phase, where actors were confronted with a juvenile conspecific in the maze for the first time. As our analysis showed, the duration of the calls decreased during the second repetition when the animals were already familiar with the context of the SSPT phase.

Last but not least, in the acoustic features’ analysis of 50‐kHz calls, we found that both groups’ peak frequency of calls in the social reward zone was significantly higher than the call frequency in the sucrose zone (Figure S2). To our knowledge, no study has yet compared the 50‐kHz call frequency in concurrent social and nonsocial reward contexts; therefore, this finding may open a new avenue for future relevant research.

### 6.2 Limitations

We only used male rats. A recent study (Uzuneser et al., 2019) reported the significant importance of sex on dopaminergic, serotoninergic, and noradrenergic changes in the dorsal striatum of tgDISC1 rats, and this study showed no change in DA levels in the dorsal striatum in male tgDISC1, which is in contrast to previous findings. This study highlights the role of considering sex in studying the DISC1 phenotype and translational research in general. Therefore, future studies using tgDISC1 rats should consider male and female rats.

In addition, in the behavioral data analysis, we only considered the time animals spent in reward zones (social and nonsocial). However, this time does not provide information about the time animals spent on specific behaviors (e.g., exploratory sniffing or rearing). In this regard, it has been shown that there is a positive correlation between highly active behaviors (jumping or playing) and specific 50‐kHz USV subtypes and a negative correlation between less active behaviors (sniffing and rearing) and 50‐kHz USV (Burke et al., 2017); therefore, analyzing certain behaviors and their association with 50‐kHz USVs could be a more efficient approach.

Because of the study design, 50‐kHz USVs in the social reward zone could be emitted by both the actor and social partner rats (Table 1). Hence, during social interaction, there were always two rats that emitted USVs, while during sucrose consumption, we measured the USVs of only one rat. Although USV source allocation was applied, it is impossible to rule out with certainty that the difference in USV call rates between the social and nonsocial reward zones also partly reflected the difference in numbers of animals emitting USVs. Therefore, the results of this study, although replicating previous results (Mulvihill & Brudzynski, 2018; Seidisarouei et al., 2021), should be interpreted with caution.

**TABLE 1:** Between‐group differences in the number of 50‐kHz calls in the different zones of the setup.

	Zone				
Group	Social reward	Sucrose reward	Neutral	Out^1^	Total
tgDISC1	7238	2845	2327	2580	14,990
WT	8060	2413	2417	3022	15,912

^1^ *Calls in the out column were vocalized outside of any of the reward or neutral zones*.

Abbreviations: tgDISC1: transgenic DISC1; WT: wild‐type.

**TABLE 2:** The result of post hoc tests on all 50‐kHz ultrasonic vocalizations (USVs)

A. Dependent variable	Group	Mean	Standard err.	*p*‐Value	
Call rate	tgDISC1	.513	.092	.771	
WT	.481	.060			
B. **Dependent variable**	**Repetition**	**Mean**	**Standard err**.	***p*‐Value**	
Call rate	First	.527	.053	.194	
Second	.467	.065			
C. **Dependent variable**	**Reward zone**	**Mean**	**Standard err**.	***p*‐Value**	
Call rate	Social	.694	.071	.001	
Sucrose	.300	.203			
D. **Dependent variable**	**Reward zone × repetition**		**Mean**	**Standard err**.	***p*‐Value**
Call rate	Social	First	.671	.062	.506
Second	.716	.091			
Sucrose	First	.382	.056	.002	
Second	.217	.048			
E. **Dependent variable**	**Group**	**Mean (s)**	**Standard err**.	***p*‐Value**	
Call duration	tgDISC1	.030	.014	.196	
WT	.027	.010			
F. **Dependent variable**	**Reward zone**	**Mean (s)**	**Standard err**.	***p*‐Value**	
Call duration	Social	.028	.008	.920	
Sucrose	.029	.001			
G. **Dependent variable**	**Repetition**	**Mean (s)**	**Standard err**.	***p*‐Value**	
Call duration	First	.030	1.2	.011	
Second	.027	1.0			
H. **Dependent variable**	**Group**	**Mean (kHz)**	**Standard Err**.	***p*‐Value**	
Call frequency	tgDISC1	58.5	.085	.597	
WT	59.3	.067			
I. **Dependent variable**	**Reward zone**	**Mean (kHz)**	**Standard err**.	***p*‐Value**	
Call frequency	Social	60.3	.008	.004	
Sucrose	57.5	.001			
J. **Dependent variable**	**Repetition**	**Mean (kHz)**	**Standard err**.	***p*‐Value**	
Call frequency	First	59.2	.909	.589	
Second	58.7	.934			

**TABLE 3:** The mixed linear model regression results; % of calls and time spent in the social reward zone

Model:	Mixed LM	Dependent variable:	% of calls in social			
No. observations:	24	Method:	REML			
No. groups:	2	Scale:	116.6			
Min. group size:	12	Log‐likelihood:	−85.4			
Max. group size:	12	Converged:	Yes			
Mean group size:	12.0					
	**Coef**.	**Std.err**.	***z***	***p* > |*z*|**	**[.025 0.975]**	
Intercept	61.026	20.169	3.026	0.002	21.49	100.5
Group [T. tgDISC1]	33.684	28.915	1.165	0.244	22.98	90.3
% Of time in social	0.271	0.305	0.374	0.374	‐0.326	0.86
% Of time in social: group [T. tgDISC1]	−0.623	0.461	−1.349	0.177	−1.527	0.28
Group var	116.658					

## 7 CONCLUSION

We recently reported social anhedonia in tgDISC1 rats. However, here, we found no group‐dependent association between social interaction and 50‐kHz USV emission. We, therefore, have no evidence to assume that 50‐kHz USVs are related to, or mediate, the DISC1 deficit in social motivation.

### PEER REVIEW

The peer review history for this article is available at https://publons.com/publon/10.1002/brb3.2984.

## Supporting information


**Supplementary Figure 1**. Mean of 50‐kHz calls over eight minutes. **A** and **B** depict the change in 50‐kHz calls per minute for both groups in the social and sucrose reward zone, respectively
**Supplementary Table 1**: Three‐way ANOVA on number 50‐kHz Calls over 8 minutes.
**Supplementary Figure 2**. The significant difference in 50‐kHz calls peak frequency between the two zones.

## Data Availability

The data that support the findings of this study are available from the corresponding author upon reasonable request.
