# Supplementary material for: 50‐kHz ultrasonic vocalizations do not signal social anhedonia in transgenic DISC1 rats
Source: Brain Behav. 2023 Apr 5;13(5):e2984. doi: 10.1002/brb3.2984 (PMC10176014; doi:10.1002/brb3.2984)
Supplement: Supplementary file 1 — Supplementary Figure 1. Mean of 50‐kHz calls over eight minutes. A and B depict the change in 50‐kHz calls per minute for both groups in the social and sucrose reward zone, respectively Supplementary Table 1: Three‐way ANOVA on number 50‐kHz Calls over 8 minutes. Supplementary Figure 2. The significant difference in 50‐kHz calls peak frequency between the two zones. [file BRB3-13-e2984-s001.docx]

The visualization and analysis of the between-group difference in all 50-kHz calls over eight minutes (supplementary Figure 1 And supplementary Table 1) and the significant difference in 50-kHz calls peak frequency between the two zones. (supplementary Figure 2).

1. **The number of 50-kHz calls.**


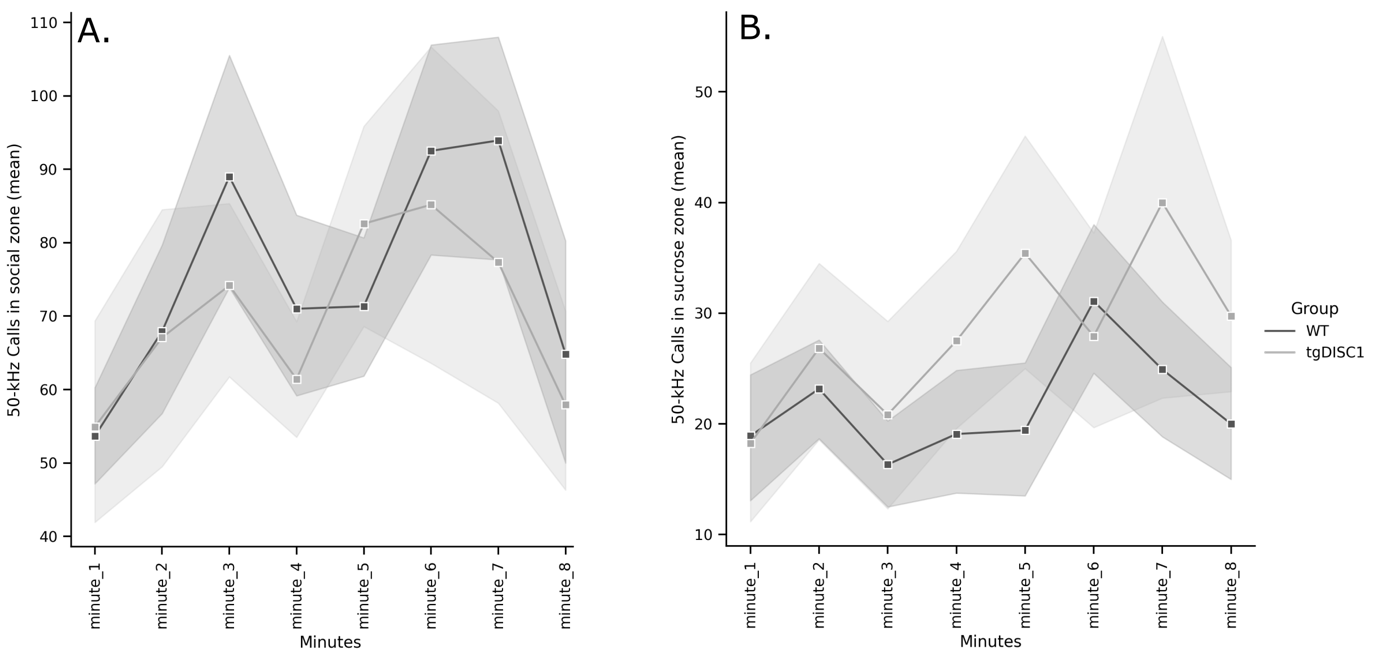


**Supplementary Figure 1.** Mean of 50-kHz calls over eight minutes. **A** and **B** depict the change in 50-kHz calls per minute for both groups in the social and sucrose reward zone, respectively. The gray shadings show the standard error.

**Supplementary Table 1:** Three-way ANOVA on number 50-kHz Calls over 8 minutes.

|  | | **df** | | **F** | | **P** | |  |  |
| --- | --- | --- | --- | --- | --- | --- | --- | --- | --- |
| Minutes |  | 7 |  |  | 3.486 |  | 0.002 | |  |
| Minutes ✻ Group |  | 7 |  |  | 0.538 |  | 0.804 | |  |
| Reward zone |  | 1 |  |  | 84.60 |  | < .001 | |  |
| Reward zone ✻ Group |  | 1 |  |  | 1.243 |  | 0.277 | |  |
| Minutes ✻ Reward zone |  | 7 |  |  | 1.099 |  | 0.366 | |  |
| Minutes ✻ Reward zone ✻Group |  | 7 |  |  | 0.641 |  | 0.366 | |  |

--------------------------------------------------------------------

|  | | **df** | | **F** | | **p** | |
| --- | --- | --- | --- | --- | --- | --- | --- |
| Group |  | 1 |  | 0.006 |  | 0.941 |  |

--------------------------------


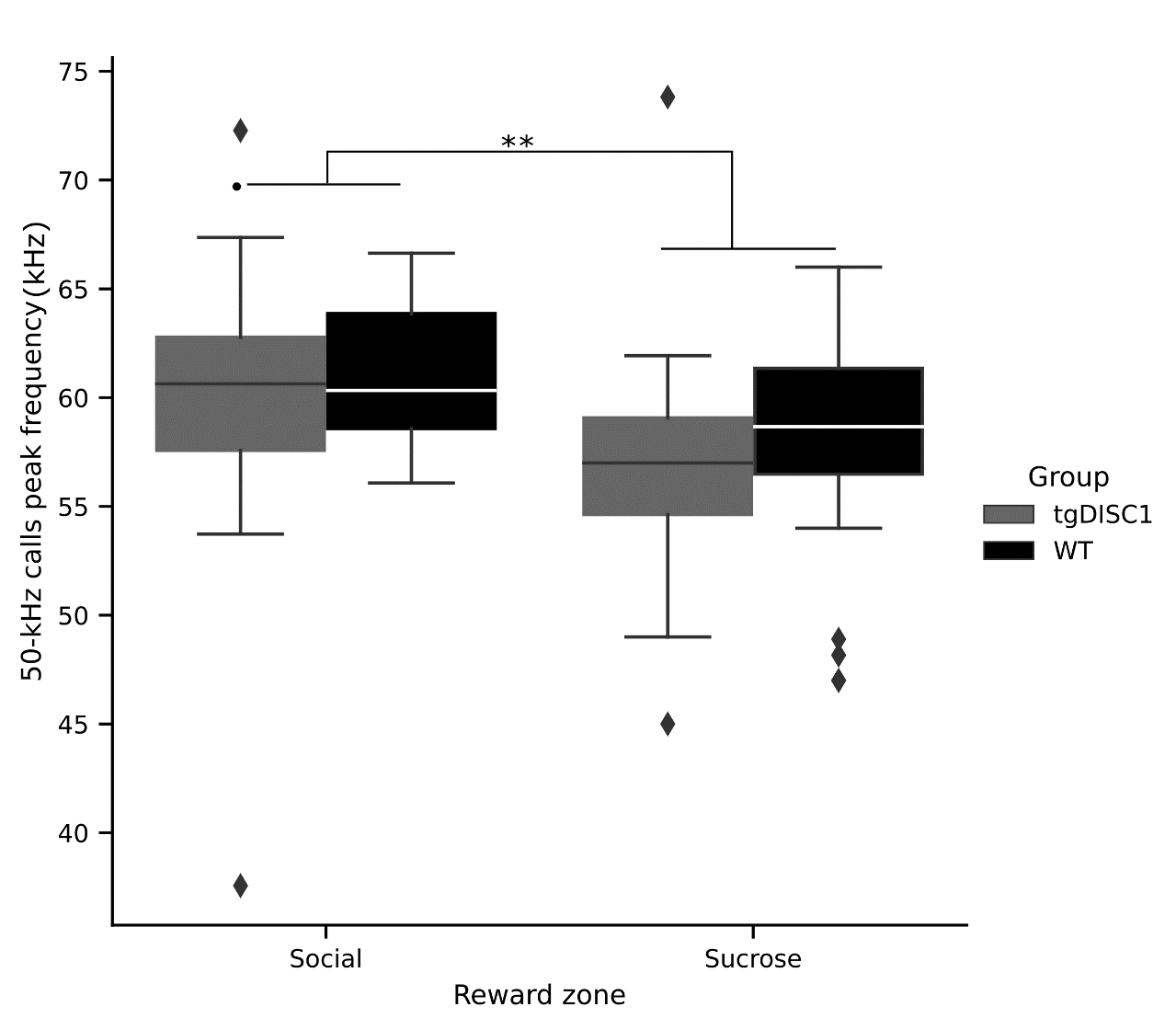


**Supplementary Figure 2**. The significant difference in 50-kHz calls peak frequency between the two zones.
